# Connect 2 Care, a Novel Community Outreach Program for Vulnerably Housed Patients With High Acute Care Use: A Mixed-Methods Study Protocol

**DOI:** 10.3389/fpubh.2021.605695

**Published:** 2021-10-08

**Authors:** Kerry A. McBrien, Van Nguyen, Dailys Garcia-Jorda, Kimberly Rondeau, Alicia Polachek, Hasham Kamran, Eddy Lang, William Ghali, Cheryl Barnabe, Ted Braun, Patrick McLane, Katrina Milaney, Paul E. Ronksley, Ginetta Salvalaggio, Eldon Spackman, Karen L. Tang, Tyler Williamson, Gabriel Fabreau

**Affiliations:** ^1^Department of Family Medicine, University of Calgary, Calgary, AB, Canada; ^2^Department of Community Health Sciences, University of Calgary, Calgary, AB, Canada; ^3^Calgary Urban Project Society, Calgary, AB, Canada; ^4^Department of Medicine, University of Calgary, Calgary, AB, Canada; ^5^Faculty of Health Sciences, Simon Fraser University, Burnaby, BC, Canada; ^6^Department of Emergency Medicine, University of Calgary, Calgary, AB, Canada; ^7^Alberta Health Services, Calgary, AB, Canada; ^8^Alberta Health Services, Edmonton, AB, Canada; ^9^Department of Family Medicine, University of Alberta, Edmonton, AB, Canada

**Keywords:** homelessness, poverty, acute care, program evaluation, health navigation, social determinants of health, transitional case management

## Abstract

**Introduction:** Vulnerably housed individuals, especially those experiencing homelessness, have higher acute care use compared with the general population. Despite available primary care and social services, many face significant challenges accessing needed services. Connect 2 Care (C2C) is a novel transitional case management program that includes registered nurses and health navigators with complementary expertise in chronic disease management, mental health and addictions, social programs, community health, and housing, financial, transportation and legal resources. C2C bridges acute care and community services to improve care coordination.

**Methods and Analysis:** We will perform a mixed-methods evaluation of the C2C program according to the Donabedian framework of *structure, process and outcome*, to understand how program structure and process, coupled with contextual factors, influence outcomes in a novel intervention. Eligible patients are homeless or unstably housed adults with complex health conditions and high acute care use. Change in emergency department visit rate 12-months after program enrolment is the primary outcome. Secondary outcomes include 12-month post-enrolment hospital admissions, cumulative hospital days, health-related quality of life, housing status, primary care attachment and substance use. Qualitative methods will explore experiences with the C2C program from multiple perspectives and an economic evaluation will assess cost-effectiveness.

**Discussion:** Academic researchers partnered with community service providers to evaluate a novel transitional case management intervention for vulnerably housed patients with high acute-care use. The study uses mixed-methods to evaluate the Connect 2 Care program according to the Donabedian framework of structure, process and outcome, including an assessment of contextual factors that influence program success. Insights gained through this comprehensive evaluation will help refine the C2C program and inform decisions about sustainability and transferability to other settings in Canada.

## Introduction

Individuals who experience housing instability are disadvantaged due to poverty and stressors such as isolation, lack of social support, and cultural or racial exclusion (1). These individuals have high morbidity and acute care use compared with the general population (2–5). Among the homeless and vulnerably housed, the probability of survival to age 75 is 32% for men and 60% for women (6). Patients who are homeless remain hospitalized longer, resulting in higher costs, especially when there is concomitant mental illness and addictions (7–11). Homelessness and poverty are significant barriers to care; often forcing individuals to prioritize food and basic needs over adherence to recommended medical care (10, 12, 13). Further, stigma and discrimination in the healthcare system can cause distrust, thus preventing collaborative engagement and leading to acute care presentation after illness severity escalates (14–16).

While primary care and social services are available, they are difficult to access and do not meet the needs of many individuals with housing instability in the community (2, 4, 12, 13). Those discharged into the community are two to four times more likely than the general population to have a repeat emergency department (ED) visit within seven days (4). Some individuals may be attached to a medical home; however, poor continuity among acute care, primary care, and social support services, impedes coordination of care and services (10, 13, 17).

Transitional case management programs can improve health and social outcomes, increase patient and staff satisfaction, and reduce acute care use (18–24). While nurse case managers provide professional multidisciplinary care coordination, community-based programs that include health (or patient) navigators (HNs) can improve access and appropriateness of care (25–30). HNs form supportive relationships with patients and serve as intermediaries between healthcare, social services and the community (29). Previous studies support the effectiveness of HNs in improving care and decreasing acute care use in complex patients; however, individual study results vary, likely due to contextual differences that may be especially important in vulnerable subpopulations (20, 23, 31, 32).

We partnered with an established urban community health center [Calgary Urban Project Society (CUPS)], a non-profit emergency shelter and housing agency (Calgary Alpha House Society), the provincial health system [Alberta Health Services (AHS)], and a provincial grant funding agency (Alberta Innovates), to develop, implement, refine and evaluate a novel transitional case management program that combines elements of intensive case management and health navigation for vulnerably housed and homeless individuals. The Connect 2 Care (C2C) program aims to improve the quality, access and coordination of care for patients with unstable housing and high acute care use. The proposed mixed-methods evaluation will provide a rigorous scientific evaluation of the C2C program.

## Methods and Analysis

### Study Setting

Calgary, Alberta, the fourth largest city in Canada, experienced significant changes leading up to and during the implementation of Connect 2 Care (33, 34). Calgary's population grew by 25% to 1,596,248 over the preceding decade (33). Following exponential growth in homelessness between 1992 and 2008 (34), Calgary's homeless population remained stable and in 2016, when we began this work, the point prevalence of homeless individuals in Calgary was estimated at 3,430 (35). At the time, the rental vacancy rate in Calgary was 7.0% and the average and median market rents were $1,150 CAD/month and $1,120 CAD/month, respectively (36). Additionally, Calgary and Alberta are facing an ongoing opioid overdose epidemic; Alberta has the second highest incidence of opioid poisonings in Canada, with substantial increases in associated ED visits and hospital admissions (37).

### The Connect 2 Care Intervention

**Figure 1:**
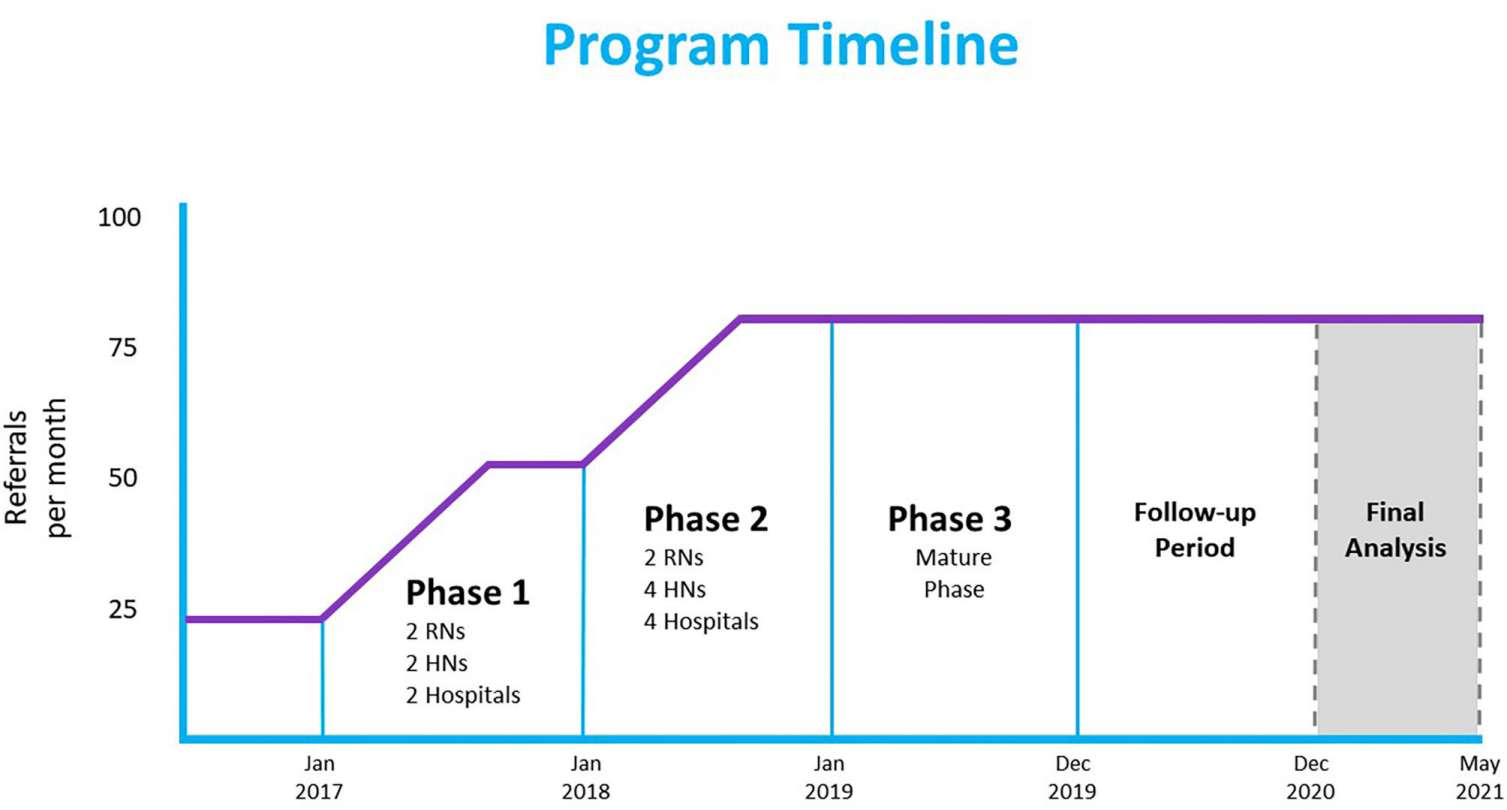
Program implementation and study timeline.

C2C is a multidisciplinary mobile outreach team that provides transitional case management, advocacy, and care navigation for socially vulnerable patients. Clinical and operational leadership at CUPS developed the C2C pilot, launched with two registered nurses (RNs) in November 2015, in partnership with Foothills Medical Center, the largest of four tertiary care hospitals in Calgary. With innovation grant funding, we expanded the program across three implementation phases (see Figure 1). In Phase 1 (January–December 2017) two HNs were added; in Phase 2 (January–December 2018) the referral base expanded to all four Calgary hospitals, as well as community agencies, using two additional HNs; in Phase 3 (January–December 2019), the program reached maturity with 2 RNs and 4 HNs. HNs are hired from a pool of emergency shelter outreach workers at Calgary Alpha House Society, a housing, shelter, outreach, and detoxification facility. HNs are acquainted with the target population's social and community context and are provided health and health system navigation training. Collectively, the C2C team has expertise in chronic disease management, mental health, harm reduction and addictions, as well as extensive knowledge of social programs, community health, housing, financial and transportation resources.

To be eligible for the program, patients must be ≥ 18 years of age, homeless or unstably housed (38), have ≥ 3 ED presentations, or ≥ 2 hospitalizations within the past year. While definitions of high acute care use vary, we chose the most conservative definition (18) in order to make the program inclusive and allow refinement to occur over the initial phases of implementation. Preliminary data gathered for the first 127 patients referred to the program before expansion indicates that, on average, C2C patients had 9 ED visits in the year prior to referral and 20% had a hospital admission. The most common reasons for admission were cellulitis, sepsis, pneumonia, substance use and other mental health diagnoses. C2C patients were more likely to be male (66%), 29% identified as Indigenous, 88% used substances and 78% were homeless. Referrals are accepted from acute care facilities, health clinics, and community agencies. Reasons for referral include attachment to primary care, housing support, connection to substance use treatment, advocacy, and discharge planning.

**Figure 2:**
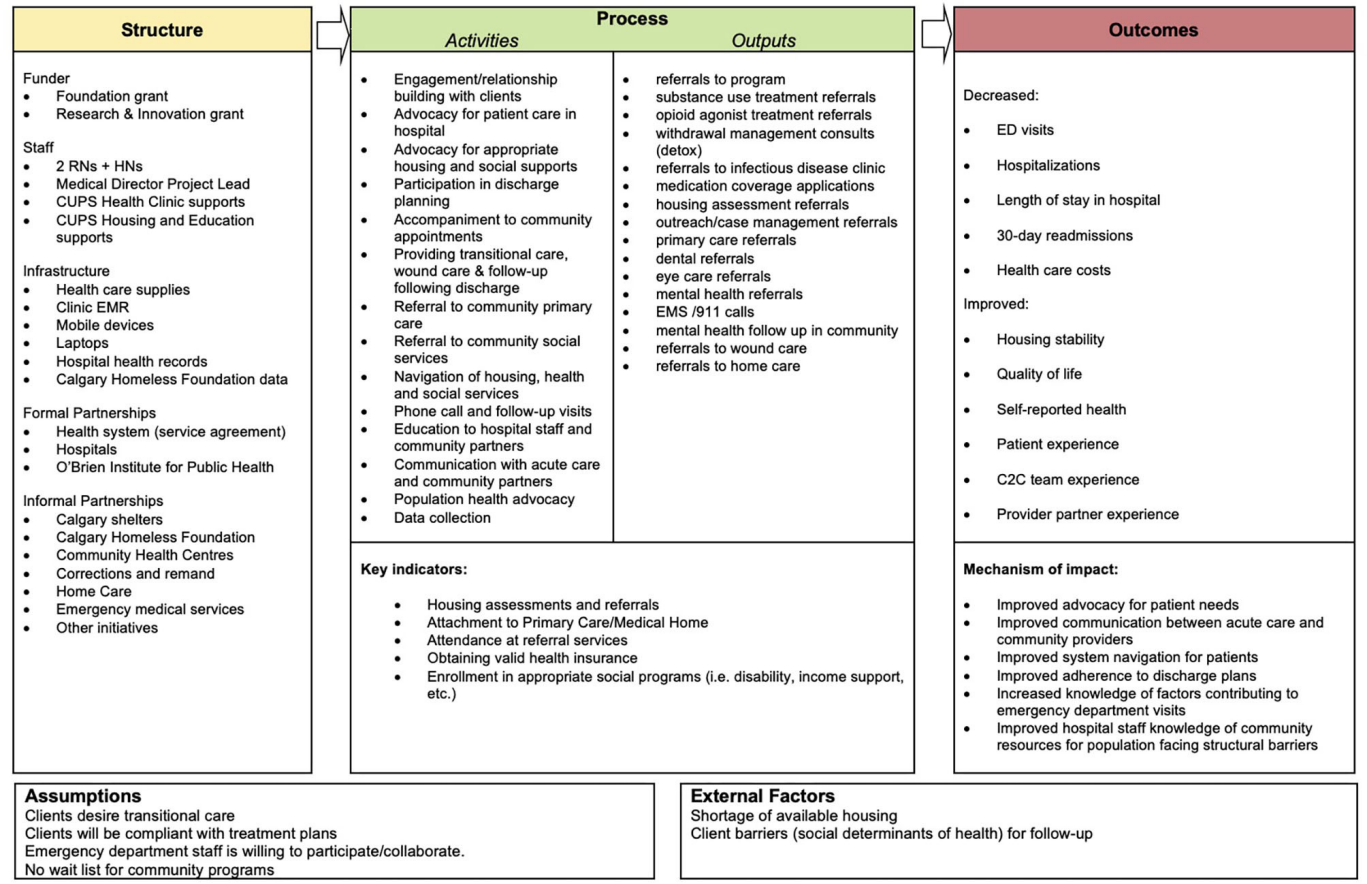
Connect 2 Care Logic Model.

C2C works closely with patients to identify their immediate needs, remove barriers to care, and coordinate the *right care at the right time*. C2C patients are assigned to either an RN or HN for case management, depending on medical complexity. The assigned navigator provides intensive case management to the patient for the duration of C2C engagement (with the goal of graduation to other programs within 6 months). The navigator meets with the patient in person to build trust and rapport, complete an intake, develop a care plan, and articulate and set longer term goals. C2C navigators regularly connect with the patient to provide on-going coaching and support to navigate day-to-day crises and challenges while building patient capacity for independence. C2C supports may include connecting patients to long-term community based primary care, providing transportation, accompaniment to appointments, advocating, coaching patients to navigate health, housing, and justice systems, providing health information, and coordinating other needed services. The outreach and mobile capabilities of C2C allow the team to support patients in community, travel between acute care sites and social service agencies, and transport and accompany patients to appointments. Figure 2 depicts the C2C logic model with further details regarding specific activities.

### Evaluation Objectives

We aim to understand how structure and process, coupled with contextual factors, lead to program outcomes in a novel transitional case management intervention.

Our study objectives are:

1. To document the structure and process of the C2C program throughout three implementation phases.
2. To determine the effectiveness of the C2C program, in light of contextual factors, in reducing acute health care use and improving patient-reported outcomes.
3. To explore patient, staff, and program partner experience with the C2C program.
4. To ascertain the cost-effectiveness of the C2C program for the publicly funded health care system.

### Study Design and Evaluation Framework

**Figure 3:**
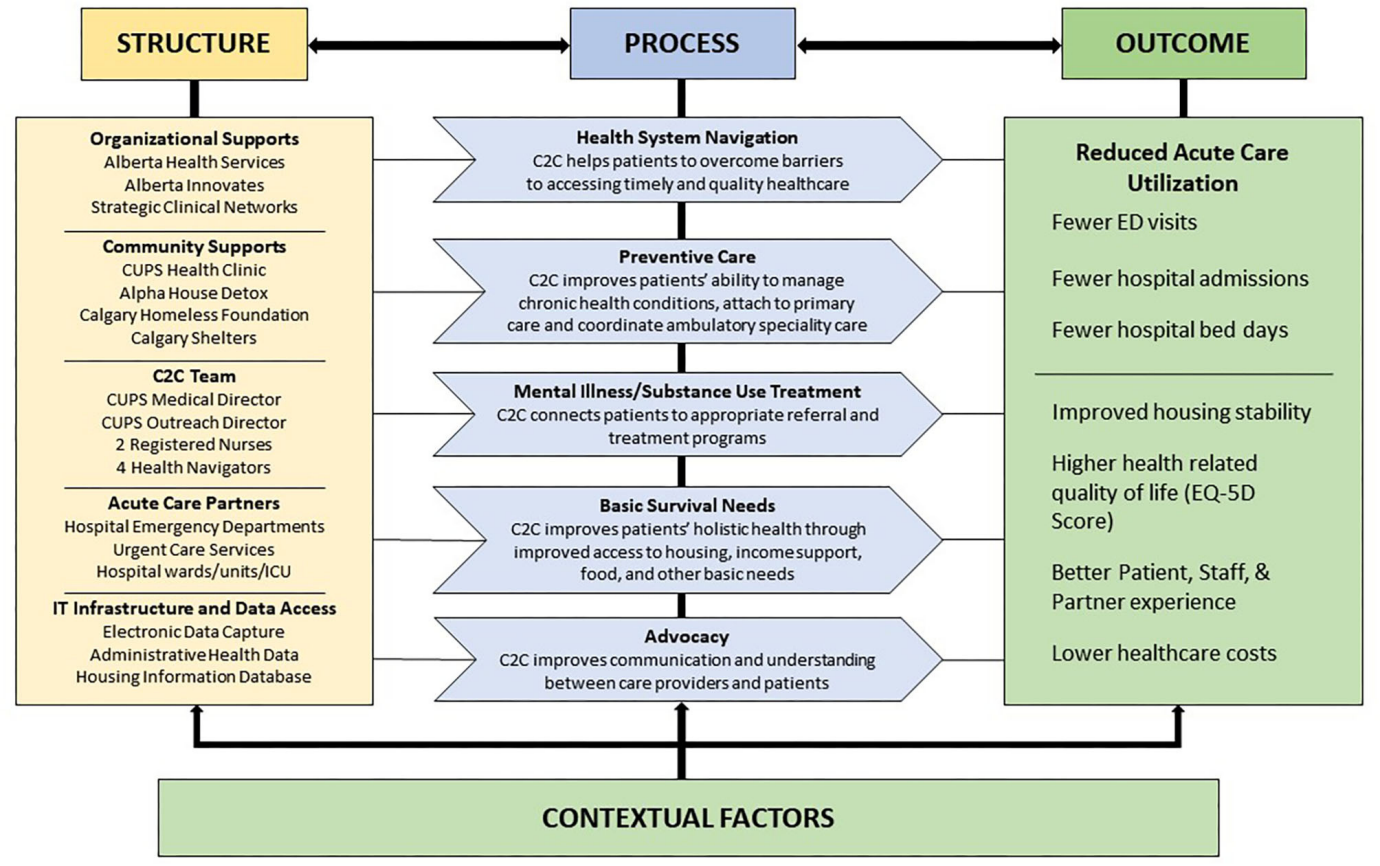
Connect 2 Care Preliminary Program Model.

We have conceptualized our evaluation using a widely accepted paradigm for measuring quality of medical care – Donabedian's model of *structure, process* and *outcome* (39). *Structure* is the setting in which care is provided and includes available resources and organizational inputs. *Process* is the means of providing and receiving care in the health system. *Outcome* is the impact of the care provided, such as the health of users within the system. The Donabedian model provides an evaluative framework based on the construct that the right organizational *structure* leads to improved *processes*, which together lead to better *outcomes* (39). Guided by the United Kingdom Medical Research Council framework for evaluating complex interventions, we extend our evaluation to also include an assessment of contextual factors that influence program success (39, 40). The current C2C program model is depicted in Figure 3.

### Structure and Process

We will document and describe the structure of C2C, as well as its processes and associated metrics. Structural elements include organizational supports, community partners, personnel, and IT infrastructure. Processes include team activities and operational procedures (e.g., program referrals and triage). Process metrics include referral numbers, engagement (i.e., patients who agree to work with C2C staff), enrolment status (i.e., active, graduated, lost to follow up, or deceased), housing stability, primary care attachment, and medication insurance acquisition. We will derive data primarily from meeting notes, program documentation, and team activity logs. To elicit further detail on structure and process, we will supplement with data from qualitative interviews and observations (see below).

### Measures of Effectiveness

#### Acute Care Use

We will use administrative health data to assess acute care use among C2C patients 12-months before and after enrolment: ED visits (primary outcome); hospital admissions; repeat ED visits within 72 h of discharge; unplanned readmissions within 30 days among hospitalized patients; total hospital bed days; deaths; ED and hospital discharge diagnoses; and ICU admissions. Data will be obtained by linking unique provincial health insurance numbers to administrative health data housed by Alberta Health Services.

#### Patient-Report Outcomes

We will collect the following patient-reported data from C2C patients at 6 and 12 months post-enrolment using in-person structured interviews comprised of closed-ended questions: health-related quality of life using the EuroQoL 5 Dimensions (EQ-5D) questionnaire (41); housing information [where they slept at night for the past 2 weeks and self-perceived stability and satisfaction (42)], primary care data (attachment [yes/no], self-perceived quality) and risk factors {drug intake [DUDIT (43)], smoking (daily/occasional/not at all), and alcohol intake [AUDIT-C (44)]}. We will also ask patients if they have achieved goals that were set at program intake and how the C2C team helped. We will obtain baseline data, including demographic characteristics, housing and income status, substance use, and patient goals from program records. In a subset of participants, we will perform a more detailed housing assessment using the Residential Time-Line Follow-Back method, to determine the number of days in a particular housing type and the number of housing transitions (38). Given the variation and complexity of the study population, we expect loss to follow-up and difficulties in collecting 6 and 12 month post-enrolment data from all engaged patients. The C2C team will utilize their strong relationships with patients and robust partnerships with community agencies and social services to retain engaged patients and locate patients for post-enrolment evaluations. In addition, C2C patients will be provided a $25 CAD VISA gift card at each instance of data collection, to compensate them for their time.

### Quantitative Data Analysis

Given the complexity of the intervention, selecting a comparison group is inherently complex. To provide statistical estimates for effectiveness, we will consider three complementary approaches: (1) a within-subject controlled analysis, (2) comparison to a cohort of similar patients, and (3) a population-level analysis assessing use of healthcare services over time. While each has interpretive caveats, they provide complementary perspectives, and together provide a clearer indication of the impact of C2C.

#### Within-Subject Comparison

In a within-subject comparison, patients serve as their own controls, eliminating the need to account for confounding due to immutable patient factors. However, observed changes in use may be due to other factors such as changes in health status over time or concomitant interventions. We will use generalized linear mixed-effects models with a negative binomial distribution to examine the ED visit rate, hospitalization rate, 30-day readmission rate and cumulative hospital bed days over the two time periods (12 months before and after enrolment), adjusting for age, sex, ethnicity, and comorbidities. We will use one-way repeated measures ANOVA to assess changes in patient-reported outcomes. We will perform stratified analyses based on phase at time of enrolment and by pre-program ED visit rate.

#### Comparison Cohort

We will identify a contemporaneous comparison cohort using administrative health data for a population not enrolled in the C2C program and who have an ED visit or hospital admission with an associated ICD-10 Z59x diagnosis (problems related to housing and economic circumstances) and at least one visit to a Calgary facility between 2014 and 2019. We will use propensity score matching to create a control group from this population. Propensity scores will be calculated for both the C2C and control cohorts and controls will be matched to C2C enrolled clients. This allows us to account for the covariates that may predict C2C enrolment, approximating random assignment and reducing selection bias between the groups. We will use a generalized linear modeling framework to estimate the effect of C2C enrolment on acute care utilization and death rates between these two groups.

#### Population-Level Analysis

Phased implementation provides an opportunity to examine trends in acute care use using administrative data for the target population as a whole. With increased reach of C2C in Calgary with each phase, the proportion of the target population exposed to C2C will increase over time, and, if successful, acute care use in this population will, on average, decrease over time. We will select a population of patients from administrative health data who present to a Calgary acute care facility and meet the following criteria: ≥ 18 years of age; ED visit or hospital admission with an associated ICD-10 Z59x diagnosis (problems related to housing and economic circumstances); ≥ 3 ED presentations, or ≥ 2 hospitalizations within the past year; and at least one high-risk health condition, as defined by ICD-10 diagnostic codes corresponding to C2C enrolment criteria.

Beginning 6 months prior to C2C program implementation, and continuing for 12 months beyond enrolment end date (i.e., May 2015–December 2020), we will retrospectively ascertain the following every 3 months: total number of patients meeting eligibility (per administrative data as above); proportion of patients currently or previously enrolled in C2C (per program data); acute care use for the prior 12 months. We will inspect the number of eligible patients enrolled in C2C over time to ensure our increased enrolment assumption is correct. We will use a generalized linear modeling framework to assess the trend over time, in both the total number of eligible patients in administrative data (to assess change in the absolute number) and average acute care use (to assess change in use among those who meet criteria), measured at 3-month intervals. We will adjust for age, sex and defined health conditions.

#### Power Calculation

We calculated power for the within-subject comparison for the primary outcome of ED visit rate over 12 months. Under ideal conditions intensive case management with outreach may reduce ED visits by 30% in high users (18). Assuming a mean pre-intervention rate of 9.3 visits/year (as noted for Phase 1 patients) and a post-intervention rate of 6.5 visits/year, a common standard deviation of 10, and a correlation between paired observations of 0.05, a sample of 257 individuals will provide more than 90% power to detect a difference at the 5% level of significance. Note that if the correlation increases, the power will increase, hence our choice of a low correlation. We have a waiver of consent to access administrative health data; therefore all patients that engage with the C2C team are eligible for inclusion in the administrative data analysis. Given our use of administrative data with full capture for participants in Alberta, any loss to follow up will be due to outmigration or invalid personal health insurance numbers. Despite this expected attrition, with 249 engaged patients in July 2018, by end of 2019, we will have an adequate study sample to detect meaningful differences.

### Patient, Staff, and Partner Experience

We will use multiple data sources to explore experience with the C2C program: semi-structured interviews of patients, C2C staff and operational leads, program partners (hospital and community agency staff who refer to and work with the C2C team), and decision makers (health system and community leaders), as well as field observations of the C2C team. Semi-structured interviews with multiple populations provide a unique opportunity to identify unexpected barriers and experiences not captured in surveys (45), while field observations provide an opportunity to observe the team's daily activities.

#### Interviews

We will conduct patient, staff and partner interviews at various time points after program launch. Purposive sampling of interview subjects will ensure broad representation of axes of social vulnerability (e.g., housing status, age, gender, and ethnicity) and personal experiences, both working within, and in partnership with C2C, respectively (46). All recruitment will occur by invitation. The final number of interviews will depend on the variability of responses, and recruitment will continue until theoretical saturation is reached (47, 48), (i.e., when no new meaningful concepts arise from the data). We anticipate we will require between 20 and 30 patient interviews and 30–40 staff and partner interviews (given the diversity across agencies) (49, 50). All interviews will be conducted in a private space (e.g., partner office) and location convenient to the participant. Interviews will be in-person or *via* telephone, semi-structured, and follow an interview guide tailored to the individual being interviewed (i.e., patient, staff, program partner or decision maker). Interviews will explore how participants interact with the program, positive and negative experiences, impact of C2C, perceptions on the services provided, and other contextual factors of influence.

#### Field Observations

A researcher will observe C2C team members during their routine workday, taking brief notes that are translated into comprehensive field notes. Recorded data will focus on team member activities, dialogue, physical spaces, interactions with clients and partners, and interactions among team members. Observations, each 3–4 h in duration, will sample different days of the week, times of day, and different team members including nurses, health navigators, and managers. While patient interactions will be the primary focus, team meetings, both formal and informal, as well as office work, will also be sampled.

### Qualitative Data Analysis

Facilitated by NVivo software, we will use thematic analysis to analyze interview transcripts and observation field notes (51). A research associate trained in qualitative methods will use data and theory-driven coding (51), to develop a preliminary codebook (52). Theory driven codes will be derived from the conceptual framework of structure, process and outcome. Data-driven codes will include emerging ideas, patterns, and concepts in the data that are not captured by the theory-driven codes. The codebook will consist of codes clustered into overarching themes and will include code names, their definitions, inclusion and exclusion criteria, and examples of text. The research team will review the preliminary codebook to discuss the interpretation and application of the codes and whether the themes capture the conceptual framework. The codebook will then be used by at least two analysts to independently code a sample of transcripts; analysts will have the ability to add new codes if they identify emerging codes. The research team will meet frequently to resolve discrepancies in coding and come to an agreement regarding the definitions of the codes and how they should be applied. The codebook will be updated as required throughout. Inter-rater reliability (IRR) tests will be performed on a sample of transcripts that have been independently coded but not yet discussed. When IRR tests reach a kappa of 360, remaining transcripts will be coded by one analyst and discussion will be restricted to new codes.

A codebook will help in managing the large amount of data from different sources (interviews and observations), promote consistency amongst coders (53), and enhance data understanding and interpretation. We will enhance the trustworthiness of our analysis through triangulation (examining data from several sources and perspectives). We will account for participants' social roles (staff, managers, patients, program partners, and decision makers), demographics, and the professional, academic and experiential backgrounds of researchers involved.

### Data Integration, Model Refinement and Understanding Across Structure, Process, and Outcome

While we will analyze quantitative and qualitative data in parallel, methodological triangulation will focus specifically on validating and refining our program model and gaining a richer understanding of how individual components are linked within context, by comparing and contrasting our findings across data sources (54, 55). To do so, we will systematically examine the components of our program model against our evaluation findings, looking for areas of confirmation, expansion or discordance between both the existing model and new findings, and across data sources. Any discordance will be explored narratively.

### Cost Effectiveness

We will perform a cost-utility analysis with a 1-year time horizon, from the perspective of the publicly funded health care system, comparing the C2C program to usual care. The analysis will follow guidance from the Canadian Agencies for Drugs and Technologies (CADTH) (56). Direct patient level health care costs will be obtained from Alberta Health Services (12 months prior to and following program enrolment): hospital admissions, ED visits, urgent care visits, ambulatory care, substance use treatment programs, medication and physician costs. Hospital costs are based on facility-specific costs per day and include direct and indirect costs such as diagnostic imaging, laboratory, housekeeping, administration, etc. Monthly total health care costs will be calculated per patient by summing across available cost data. Program costs will be obtained from the operational budget: wages, training, transportation, management, and IT costs. Health outcomes of interest will be lives lost and the change in health-related quality of life (measured by the EQ-5D). Utility will be derived from EQ-5D data using Canadian based preferences (57). A linear extrapolation of utilities over time will be used to calculate per patient quality-adjusted life years (QALYs).

#### Cost Analysis

A generalized linear model with random effects controlling for time and patient characteristics such as age and sex will be used to estimate mean health care costs pre- and post-enrolment (58). The difference in health care costs between the pre- and post-enrolment periods will be calculated and compared to program costs.

#### Cost-Effectiveness

The incremental cost per additional QALYs will be calculated. To jointly estimate the effect of the C2C program on costs and QALYs we will use a seemingly unrelated regression analysis (59), controlling for patient-level characteristics. We will use estimated costs and QALYs to calculate the incremental cost-effectiveness ratio of the C2C program, which will be compared to commonly accepted cost-effectiveness thresholds. Cost-effectiveness acceptability curves will be used to represent the model uncertainty (60).

## Discussion

The proposed study has some limitations. First is the absence of a randomly allocated comparison group. The use of propensity score matching to create a control group will help to reduce bias but unmeasured confounding by variables that are not available in administrative health data may occur. Second, the nature of our study population may lead to challenges in locating and connecting with patients for interviews and follow-up. Therefore, we may not capture the unique experiences of patients facing additional barriers that impact ability and willingness to connect. Our follow-up period also limits our ability to determine whether outcomes persist or new outcomes arise past 12 months post-enrolment. However, a longer follow-up period would create additional challenges in connecting with patients and increase bias within our results. Third, results might be influenced by time-varying confounders unrelated to C2C, such as natural changes in health. Finally, our evaluation occurs within the context of a single city which reduces generalizability, thus careful consideration should be given to the local context when applying evaluation findings across settings.

Despite these limitations, our study has several advantages. A main advantage is our mixed-methods approach, which examines the impact of the program both quantitatively, across multiple outcomes, and qualitatively. Our qualitative data include observations and interviews with multiple stakeholder groups. Integration of data across multiple sources will ensure that valuable patient, staff, and partner perspectives are considered. As our evaluation occurs within the context of the C2C program, results will also reflect patient and program outcomes in a real-world setting. Thus, results will directly inform decisions on C2C and related interventions.

C2C is an innovative community outreach program that combines elements of case management and health navigation to bridge the divide between acute care and community-based services for individuals with unstable housing and high acute care use. It represents a novel partnership between community-based service organizations, an academic institution, and a provincial health system. The proposed mixed-methods study will inform program sustainability and scale-up and enable translation of core elements to other urban settings. Key components of the C2C program model – community agency engagement, use of HNs to expand reach and scope, and partnership with the health care authority – are all transferable, though the exact nature of programming will depend on the local environment and must be integrated with existing initiatives. In addition to guiding policy within Alberta, the results of this multi-faceted research project will be applicable to other healthcare organizations, both within Canada and internationally.

## Ethics Statement

This study was approved by the University of Calgary Conjoint Health Research Ethics Board (reference ID: REB16-0896). Informed written consent will be obtained for all primary data collection activities: structured and semi-structured interviews, and observations. We have a waiver of consent to access administrative health data. Insights gained through this comprehensive evaluation will help refine the C2C program and inform decisions about sustainability and transferability to other settings in Canada.

## Author Contributions

KAM, VN, EL, and GF co-designed the intervention in partnership with operational stakeholders, with input from TB. KAM, GF, and WG conceived the study, with input from DG-J, KR, AP, HK, CB, PM, KM, PR, ES, KT, and TW. KAM and GF drafted the protocol. All authors contributed to revisions and approved the protocol.

## Funding

This work was supported by Alberta Innovates through a Partnership for Research and Innovation in the Health System (Grant No. 201600428), the Canadian Institutes of Health Research (Grant No. PJT 165998), and the O'Brien Institute for Public Health at the University of Calgary.

## Conflict of Interest

The authors declare that the research was conducted in the absence of any commercial or financial relationships that could be construed as a potential conflict of interest.

## Publisher's Note

All claims expressed in this article are solely those of the authors and do not necessarily represent those of their affiliated organizations, or those of the publisher, the editors and the reviewers. Any product that may be evaluated in this article, or claim that may be made by its manufacturer, is not guaranteed or endorsed by the publisher.

## Abbreviations

C2C: Connect 2 Care
ED: Emergency department
HNs: Health navigators
CUPS: Calgary Urban Project Society
AHS: Alberta Health Services
RNs: Registered nurses
EQ-5D: EuroQoL 5 Dimensions questionnaire
IRR: Inter-rater reliability
QALYs: quality-adjusted life years
CHREB: Conjoint Health Research Ethics Board.

## References

[1] Baker D Mead N Campbell S. Inequalities in morbidity and consulting behaviour for socially vulnerable groups. Br J Gen Pract. (2002) 52:124–30. 11885821 PMC1314218

[2] Chan BT Ovens HJ. Frequent users of emergency departments. Do they also use family physicians' services? Can Fam Phys Medecin Fam Can. (2002) 48:1654–60. 12449550 PMC2213944

[3] Geurts J Palatnick W Strome T Sutherland KA Weldon E. Frequent users of an inner-city emergency department. CJEM. (2012) 14:306–13. 10.2310/8000.2012.120670 22967698

[4] Ku BS Scott KC Kertesz SG Pitts SR. Factors associated with use of urban emergency departments by the U.S. homeless population. Public Health Rep. (2010) 125:398–405. 10.1177/003335491012500308 20433034 PMC2848264

[5] Kushel MB Perry S Bangsberg D Clark R Moss AR. Emergency department use among the homeless and marginally housed: results from a community-based study. Am J Public Health. (2002) 92:778–84. 10.2105/AJPH.92.5.778 11988447 PMC1447161

[6] Hwang SW Wilkins R Tjepkema M O'Campo PJ Dunn JR. Mortality among residents of shelters, rooming houses, and hotels in Canada: 11 year follow-up study. BMJ. (2009) 339:b4036. 10.1136/bmj.b4036 19858533 PMC2767481

[7] Hwang SW Weaver J Aubry T Hoch JS. Hospital costs and length of stay among homeless patients admitted to medical, surgical, and psychiatric services. Med Care. (2011) 49:350–4. 10.1097/MLR.0b013e318206c50d 21368678

[8] Latimer EA Rabouin D Cao Z Ly A Powell G Aubry T . Costs of services for homeless people with mental illness in 5 Canadian cities: a large prospective follow-up study. CMAJ Open. (2017) 5:E576–E85. 10.9778/cmajo.20170018 28724726 PMC5621955

[9] Vandyk AD Harrison MB VanDenKerkhof EG Graham ID Ross-White A. Frequent emergency department use by individuals seeking mental healthcare: a systematic search and review. Arch Psychiatr Nurs. (2013) 27:171–8. 10.1016/j.apnu.2013.03.001 23915694

[10] Levy BD O'Connell JJ. Health care for homeless persons. N Engl J Med. (2004) 350:2329–32. 10.1056/NEJMp038222 15175433

[11] Hudon C Sanche S Haggerty JL. Personal characteristics and experience of primary care predicting frequent use of emergency department: a prospective cohort study. PLoS ONE. (2016) 11:e0157489. 10.1371/journal.pone.0157489 27299525 PMC4907452

[12] Hunter CE Palepu A Farrell S Gogosis E O'Brien K Hwang SW. Barriers to prescription medication adherence among homeless and vulnerably housed adults in three canadian cities. J Prim Care Community Health. (2015) 6:154–61. 10.1177/2150131914560610 25428404

[13] Kangovi S Barg FK Carter T Long JA Shannon R Grande D. Understanding why patients of low socioeconomic status prefer hospitals over ambulatory care. Health Affairs (Project Hope). (2013) 32:1196–203. 10.1377/hlthaff.2012.0825 23836734

[14] Fine AG Zhang T Hwang SW. Attitudes towards homeless people among emergency department teachers and learners: a cross-sectional study of medical students and emergency physicians. BMC Med Educ. (2013) 13:112. 10.1186/1472-6920-13-112 23968336 PMC3765267

[15] Kangovi S Mitra N Grande D White ML McCollum S Sellman J . Patient-centered community health worker intervention to improve posthospital outcomes: a randomized clinical trial. JAMA Intern Med. (2014) 174:535–43. 10.1001/jamainternmed.2013.14327 24515422

[16] Thornicroft G Rose D Kassam A. Discrimination in health care against people with mental illness. Intern Rev Psychiatry (Abingdon, England). (2007) 19:113–22. 10.1080/09540260701278937 17464789

[17] Berkowitz SA Hulberg AC Hong C Stowell BJ Tirozzi KJ Traore CY . Addressing basic resource needs to improve primary care quality: a community collaboration programme. BMJ Qual Saf. (2016) 25:164–72. 10.1136/bmjqs-2015-004521 26621916

[18] Althaus F Paroz S Hugli O Ghali WA Daeppen JB Peytremann-Bridevaux I . Effectiveness of interventions targeting frequent users of emergency departments: a systematic review. Ann Emerg Med. (2011) 58:41–52.e42. 10.1016/j.annemergmed.2011.03.007 21689565

[19] Bodenmann P Velonaki VS Griffin JL Baggio S Iglesias K Moschetti K . Case Management may reduce emergency department frequent use in a universal health coverage system: a randomized controlled trial. J Gen Intern Med. (2017) 32:508–15. 10.1007/s11606-016-3789-9 27400922 PMC5400747

[20] Hudon C Chouinard MC Lambert M Dufour I Krieg C. Effectiveness of case management interventions for frequent users of healthcare services: a scoping review. BMJ Open. (2016) 6:e012353. 10.1136/bmjopen-2016-012353 27687900 PMC5051491

[21] Kumar GS Klein R. Effectiveness of case management strategies in reducing emergency department visits in frequent user patient populations: a systematic review. J Emerg Med. (2013) 44:717–29. 10.1016/j.jemermed.2012.08.035 23200765

[22] Moe J Kirkland SW Rawe E Ospina MB Vandermeer B Campbell S . Effectiveness of interventions to decrease emergency department visits by adult frequent users: a systematic review. Acad Emerg Med. (2017) 24:40–52. 10.1111/acem.13060 27473387

[23] Raven MC Kushel M Ko MJ Penko J Bindman AB. The effectiveness of emergency department visit reduction programs: a systematic review. Ann Emerg Med. (2016) 68:467–83.e15. 10.1016/j.annemergmed.2016.04.015 27287549

[24] Shumway M Boccellari A O'Brien K Okin RL. Cost-effectiveness of clinical case management for ED frequent users: results of a randomized trial. Am J Emerg Med. (2008) 26:155–64. 10.1016/j.ajem.2007.04.021 18272094

[25] Balaban RB Galbraith AA Burns ME Vialle-Valentin CE Larochelle MR Ross-Degnan D . Patient navigator intervention to reduce hospital readmissions among high-risk safety-net patients: a randomized controlled trial. J Gen Intern Med. (2015) 30:907–15. 10.1007/s11606-015-3185-x 25617166 PMC4471016

[26] Balaban RB Zhang F Vialle-Valentin CE Galbraith AA Burns ME Larochelle MR . Impact of a patient navigator program on hospital-based and outpatient utilization over 180 days in a safety-net health system. J Gen Intern Med. (2017) 32:981–9. 10.1007/s11606-017-4074-2 28523476 PMC5570741

[27] Enard KR Ganelin DM. Reducing preventable emergency department utilization and costs by using community health workers as patient navigators. J Healthcare Manag Am Coll Healthcare Execut. (2013) 58:412–27; discussion 28. 10.1097/00115514-201311000-00007 24400457 PMC4142498

[28] Kelly E Duan L Cohen H Kiger H Pancake L Brekke J. Integrating behavioral healthcare for individuals with serious mental illness: a randomized controlled trial of a peer health navigator intervention. Schizophr Res. (2017) 182:135–41. 10.1016/j.schres.2016.10.031 27793514

[29] Kwan JL Morgan MW Stewart TE Bell CM. Impact of an innovative inpatient patient navigator program on length of stay and 30-day readmission. J Hosp Med. (2015) 10:799–803. 10.1002/jhm.2442 26259201

[30] Nossel IR Lee RJ Isaacs A Herman DB Marcus SM Essock SM. Use of peer staff in a critical time intervention for frequent users of a psychiatric emergency room. Psychiatr Serv (Washington, DC). (2016) 67:479–81. 10.1176/appi.ps.201500503 26766759

[31] de Vet R van Luijtelaar MJ Brilleslijper-Kater SN Vanderplasschen W Beijersbergen MD Wolf JR. Effectiveness of case management for homeless persons: a systematic review. Am J Public Health. (2013) 103:e13–26. 10.2105/AJPH.2013.301491 23947309 PMC3780754

[32] Van den Heede K Van de Voorde C. Interventions to reduce emergency department utilisation: a review of reviews. Health Policy (Amsterdam, Netherlands). (2016) 120:1337–49. 10.1016/j.healthpol.2016.10.002 27855964

[33] Statistics Canada. Population Estimates. Economic Region (2016). 10.25318/1710013701-eng

[34] Kneebone R Bell M Jackson N Jadidzadeh A. Who are the homeless? Numbers, trends and characteristics of those without homes in Calgary. Sch Public Policy Public. (2015) 8:1–16. Available online at: https://www.policyschool.ca/wp-content/uploads/2016/03/who-are-homeless-kneebone-bell-jacksonjadidzadeh.pdf

[35] Campbell R Falvo N Smith M. Calgary Fall 2016 Point-in-time Count Report. Calgary Homeless Foundation (2016).

[36] Canada Mortgage and Housing Corporation (CMHC). Rental Market Survey: Government of Canada. (2016). Available online at: https://www03.cmhc-schl.gc.ca/hmip-pimh/en/TableMapChart/RmsMethodology (accessed May 21, 2021).

[37] Canadian Institute for Health Information Canadian Centre on Substance Abuse. Hospitalizations and Emergency Department Visits Due to Opioid Poisoning in Canada. Ottawa, ON: CIHI (2016).

[38] Tsemberis S McHugo G Williams V Hanrahan P Stefancic A. Measuring homelessness and residential stability: the residential time-line follow-back inventory. J Community Psychol. (2007) 35:29–42. 10.1002/jcop.20132

[39] Donabedian A. Evaluating the quality of medical care. Milbank Q. (2005) 83:691–729. 10.1111/j.1468-0009.2005.00397.x 16279964 PMC2690293

[40] Craig P Dieppe P Macintyre S Michie S Nazareth I Petticrew M. Developing and evaluating complex interventions: the new Medical Research Council guidance. BMJ. (2008) 337:a1655. 10.1136/bmj.a1655 18824488 PMC2769032

[41] Lamers LM Bouwmans CA van Straten A Donker MC Hakkaart L. Comparison of EQ-5D and SF-6D utilities in mental health patients. Health Econ. (2006) 15:1229–36. 10.1002/hec.1125 16625671

[42] Salvalaggio G Dong KA Hyshka E Nixon L Lavergne KJ Nichols J . Enhanced multidisciplinary care for inner city patients with high acute care use: study protocol. Can J Addict. (2016) 7:34–41. 10.1097/02024458-201609000-00005

[43] Voluse AC Gioia CJ Sobell LC Dum M Sobell MB Simco ER. Psychometric properties of the drug use disorders identification test (DUDIT) with substance abusers in outpatient and residential treatment. Addict Behav. (2012) 37:36–41. 10.1016/j.addbeh.2011.07.030 21937169

[44] Bush K Kivlahan DR McDonell MB Fihn SD Bradley KA. The AUDIT alcohol consumption questions (AUDIT-C): an effective brief screening test for problem drinking. Ambulatory care quality improvement project (ACQUIP) alcohol use disorders identification test. Arch Intern Med. (1998) 158:1789–95. 10.1001/archinte.158.16.1789 9738608

[45] Boyce C Neale P. Conducting In-Depth Interviews: A Guide for Designing and Conducting In-Depth Interviews for Evaluation Input. Watertown, MA: Pathfinder International (2006).

[46] Devers KJ Frankel RM. Study design in qualitative research - 2: sampling and data collection strategies. Educ Health. (2000) 13:263–71. 10.1080/13576280050074543 14742088

[47] Green J Thorogood N. Qualitative Methods for Health Research. 3rd ed. London: Sage Publications Ltd. (2014). p. 360.

[48] Hennink MM Kaiser BN Marconi VC. Code saturation versus meaning saturation: how many interviews are enough? Qual Health Res. (2017) 27:591–608. 10.1177/1049732316665344 27670770 PMC9359070

[49] Glaser BG Strauss A. The Discovery of Grounded Theory: Strategies for Qualitative Research. Chicago, IL: Aldine (1967).

[50] Guest G Bunce A Johnson L. How many interviews are enough? An experiment with data saturation and variability. Field Methods. (2006) 18:59–82. 10.1177/1525822X05279903

[51] Boyatzis RE. Transforming Qualitative Information: Thematic Analysis and Code Development. Thousand Oaks CA: SAGE Publications Ltd. (1998).

[52] Crabtree BF Miller WL. A Template Approach to Text Analysis: Developing and Using Codebooks. Doing Qualitative Research. Newbury Park, CA: SAGE Publications Ltd. (1992). p. 93–109.

[53] DeCuir-Gunby JT Marshal PL McCulloch AW. Developing and using a codebook for the analysis of interview data: an example from a professional development research project. Field Methods. (2010) 23:136–55. 10.1177/1525822X10388468

[54] Creswell JW Klassen AC Clark VP Smith KC. Best practices for mixed methods research in the health sciences. Off Behav Soc Sci Res Natl Inst Health. (2011) 1–37. 10.1037/e566732013-001

[55] Howe KR. Mixed methods, triangulation, and causal explanation. J Mix Methods Res. (2012) 6:89–96. 10.1177/1558689812437187

[56] Guidelines for the Economic Evaluation of Health Technologies: Canada. 4th ed. Ottawa, ON: CADTH (2017). Available online at: https://www.cadth.ca/guidelines-economic-evaluationhealth-technologies-canada-0 (accessed on September 21, 2021).

[57] Bansback N Tsuchiya A Brazier J Anis A. Canadian valuation of EQ-5D health states: preliminary value set and considerations for future valuation studies. PLoS ONE. (2012) 7:e31115. 10.1371/journal.pone.0031115 22328929 PMC3273479

[58] Blough DK Ramsey SD. Using generalized linear models to assess medical care costs. Heal Serv Outcomes Res Methodol. (2000) 1:185–202. 10.1023/A:101259712366724991334

[59] Willan AR Lin DY Manca A. Regression methods for cost-effectiveness analysis with censored data. Stat Med. (2005) 24:131–45. 10.1002/sim.1794 15515137

[60] Ramsey7 S Willke R Briggs A Brown R Buxton M Chawla A . Good research practices for cost-effectiveness analysis alongside clinical trials: the ISPOR RCT-CEA Task Force report. Value Health. (2005) 8:521–33. 10.1111/j.1524-4733.2005.00045.x 16176491

